# Translocation of an Anteater (*Tamandua tetradactyla*) Infected with Rabies from Virginia to Tennessee Resulting in Multiple Human Exposures, 2021

**DOI:** 10.15585/mmwr.mm7115a1

**Published:** 2022-04-15

**Authors:** Heather N. Grome, Jane Yackley, Dilani Goonewardene, Andrew Cushing, Marcy Souza, Ariel Carlson, Linden Craig, Bryan Cranmore, Ryan Wallace, Lillian Orciari, Michael Niezgoda, Satheshkumar Panayampalli, Crystal Gigante, Mary-Margaret Fill, Timothy Jones, William Schaffner, John Dunn

**Affiliations:** ^1^Epidemic Intelligence Service, CDC; ^2^Communicable and Environmental Diseases and Emergency Preparedness Division, Tennessee Department of Health; ^3^College of Veterinary Medicine, University of Tennessee, Knoxville, Tennessee; ^4^Division of High-Consequence Pathogens and Pathology, National Center for Emerging and Zoonotic Infectious Diseases, CDC; ^5^Department of Health Policy, Vanderbilt University School of Medicine, Nashville, Tennessee.

On August 16, 2021, the Tennessee Department of Health (TDH) was notified of a positive rabies test result from a South American collared anteater (*Tamandua tetradactyla*) in Washington County, Tennessee. Tamanduas, or lesser anteaters, are a species of anteater in which rabies has not previously been reported. The animal was living at a Tennessee zoo and had been recently translocated from a zoo in Virginia. TDH conducted an investigation to confirm the rabies result, characterize the rabies variant, and ascertain an exposure risk assessment among persons who came into contact with the tamandua. Risk assessments for 22 persons were completed to determine the need for rabies postexposure prophylaxis (rPEP); rPEP was recommended for 13 persons, all of whom agreed to receive it. Using phylogenetic results of the virus isolated from the tamandua and knowledge of rabies epidemiology, public health officials determined that the animal was likely exposed to wild raccoons present at the Virginia zoo. This report describes expansion of the wide mammalian species diversity susceptible to rabies virus infection and summarizes the investigation, highlighting coordination among veterinary and human public health partners and the importance of preexposure rabies vaccination for animal handlers and exotic zoo animals.

## Case Report

**FIGURE 1:**
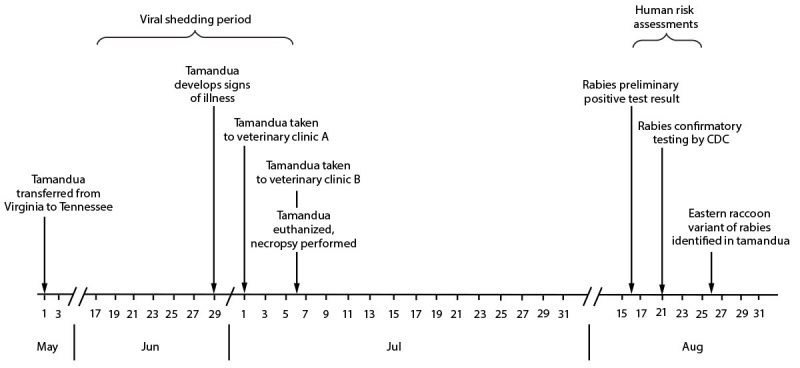
Timeline for public health investigation of a rabid tamandua (anteater) translocated from Virginia to Tennessee, May–August 2021

In early May 2021, a tamandua was translocated from a drive-through zoo in Virginia (where animals can be viewed from visitors’ vehicles) to a zoo in Washington County, Tennessee (Figure 1), where it was kept in an indoor habitat with one other tamandua and isolated from zoo visitors and wildlife. The tamanduas were not permitted out of the enclosure, and no known exposures to other animals occurred.

On June 29, the tamandua began exhibiting signs of illness including lethargy, anorexia, and diarrhea. A local veterinarian and veterinary technician at veterinary clinic A examined the tamandua on July 1. The animal was treated empirically with an antibiotic for presumed infection and vitamin K injections and returned to the zoo. After progression of clinical signs, including copious salivation, the animal was transported on July 6 to clinic B at a nearby veterinary medical college, where it was examined by a veterinarian, veterinary residents, interns, students, and a visiting veterinary consultant. Rabies was not considered in the differential diagnosis at this time because 1) there was no known bite exposure, 2) rabies had never been reported in a tamandua, and 3) the low basal body temperature of tamanduas (91°F [32.8°C]) was believed to contribute to decreased susceptibility to rabies virus infection. Routine diagnostics failed to reveal a primary cause, and supportive care was unsuccessful in improving the animal’s condition, necessitating euthanasia on July 6.

**FIGURE 2:**
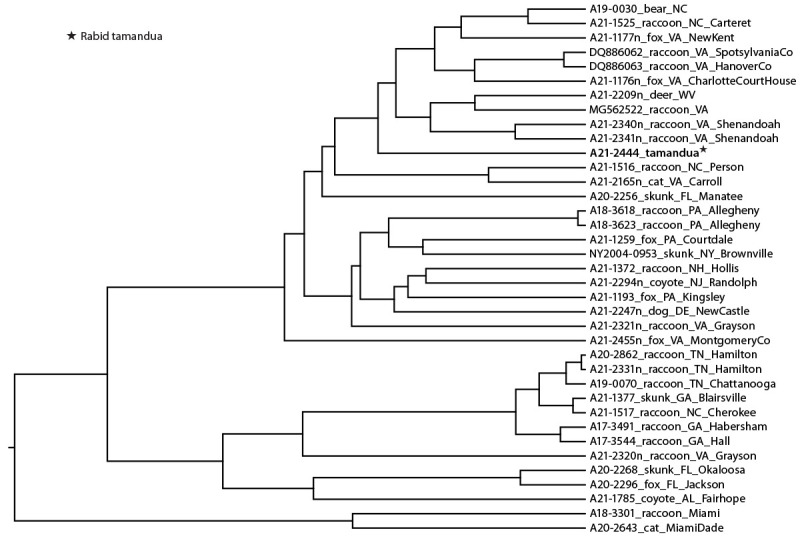
Phylogenetic analysis of rabies virus nucleoprotein gene from the rabid tamandua* identified in Tennessee with raccoon rabies virus variant sequences^†^ from Tennessee, Virginia, and other nearby states, 2021 * Specimen labeled A21-2444 was collected from the rabid tamandua. This specimen clustered with rabies virus sequences from the northeast and mid-Atlantic regions and is separate from specimens from the southeast. ^†^ Branch length is related to the number of nucleotide substitutions. The more substitutions, the longer the branch.

Necropsy, including removal of brain tissue using an electric oscillating saw, was completed at the veterinary medical college. Laboratory gowns and latex gloves were used in the necropsy suite; no additional personal protective equipment, such as eye and respiratory protection, was used. Brain tissue was submitted to an academic laboratory for histopathology. The head was not submitted to the state public health laboratory; therefore, no fresh brain material was available for rabies testing. The academic laboratory reported a preliminary positive rabies result by immunohistochemistry test on August 16, approximately 6 weeks after euthanasia. The process was not expedited because rabies was not in the differential diagnosis at time of death. TDH was notified of the positive test result, fixed brain tissue was requested, and it was submitted to CDC for confirmatory rabies testing. On August 21, rabies virus antigen was confirmed in the brain of the tamandua by immunohistochemistry and by reverse transcription–polymerase chain reaction assay (*1*). On August 26, molecular characterization determined that the rabies virus was most similar to the rabies virus variant (RVV) observed in raccoons in the eastern United States and reference sequences from Virginia. RVV was divergent from all available sequences from Tennessee, suggesting that rabies infection occurred while the animal was at the Virginia zoo (Figure 2).

## Public Health Investigation

TDH developed an assessment tool to identify persons potentially exposed to the tamandua during the rabies viral shedding period, defined as 14 days before onset of clinical signs (June 16) through the date of death (July 6) (*2*) or involvement in necropsy after death. All 22 persons identified with potential exposure completed the risk assessment. rPEP was recommended to 13 persons for nonbite exposures to the animal’s tongue and saliva (tamanduas do not have teeth). Seven persons received this recommendation either because of known or presumed exposure to saliva or because of the inability to determine if saliva was introduced to a scratch or open skin wound. Six persons received this recommendation because of potential exposure attributable to aerosolization of brain tissue, because barrier protection was limited to latex gloves and laboratory gowns during removal of brain tissue using an oscillating saw; rPEP was recommended to persons who operated the saw, other persons <10 feet from the saw, and anyone not confident of where they were in the room when the calvarium was breached. Among the 13 persons for whom rPEP was recommended, all agreed to receive it. No human rabies cases have been reported to date. This activity was reviewed by CDC and was conducted consistent with applicable federal law and CDC policy.*

The other tamandua at the Tennessee zoo enclosure was presumed to be unvaccinated because rabies vaccination records could not be located. This animal received rabies vaccine, and the zoo owner was advised to strictly quarantine it for 6 months, in concordance with the 2016 Compendium of Animal Rabies Prevention and Control (*2*). The Virginia zoo was notified regarding concerns about rabid raccoons on the property. The owner of this zoo confirmed that native wildlife was present inside the fencing perimeter. As of April 1, 2022, no additional cases of rabies related to this tamandua were identified in Virginia or in Tennessee.

## Discussion

This case demonstrates the possibility of rabies translocation by human movement of captive mammals, including species in which rabies has not been previously reported. In the United States, multiple RVVs exist in wild mammalian reservoir populations. Except for bat RVVs, distinct variants associated with major animal reservoir species occur in geographically distinct regions where transmission is mainly among members of the same species (*3*,*4*). The complete genome sequence of rabies virus isolated from this tamandua was similar to that of the eastern raccoon RVV reference sequences from Virginia, which is consistent with the presence of native wildlife (including raccoons) inside the fencing perimeter at the Virginia zoo. The eastern raccoon RVV is enzootic in 18 states and the District of Columbia (*3*). Washington County, Tennessee, has enzootic north-central skunk RVV, but this raccoon RVV is not considered enzootic in the county; no cases of the raccoon RVV have been reported in the county during the previous 5 years (*5*). Phylogenetic data and epidemiologic evidence were used to rule out local transmission and expansion of raccoon RVV into this Tennessee county, which confirmed that extensive mitigation actions were not required (*6*). Although the National Association of State Public Health Veterinarians recommends that dogs, cats, ferrets, and horses be vaccinated against rabies before interstate movement (*2*), no similar recommendations for vaccination of other captive animals are in effect. Expansion of rabies zones in the United States through translocation has substantial adverse public health implications (*7*), including threatening the health of humans, domestic animals, and other wildlife; and potentially requiring changes in wildlife rabies control measures.

Rabies detection in animals in the United States is dependent on the public health and veterinary laboratories that routinely perform rabies testing with standardized methods. The national case definition for animal rabies requires laboratory confirmation with either a positive result for the direct fluorescent antibody test or isolation of rabies virus (*8*). Timely action is required when rabies is suspected and an animal or human rabies exposure has occurred. In this situation, >1 month had lapsed between the necropsy and confirmatory diagnosis performed by CDC. Delays in appropriate diagnostic testing for rabies after necropsy caused delays in administering rPEP and inadvertently placed persons at increased risk for rabies.

Captive mammals maintained in exhibits or zoological parks typically are not completely excluded from rabies host species and can become infected. All employees who work with animals in areas where rabies is endemic should receive preexposure rabies vaccination in accordance with recommendations of the Advisory Committee on Immunization Practices (*2*,*9*). Three employees at the Tennessee zoo and veterinary staff members in this case had not received rabies preexposure vaccination, despite living in a skunk rabies reservoir area and routinely working with animals. These persons were recommended to receive rabies immune globulin and the 4-dose rPEP vaccination series after risk assessment (*10*). This case also highlights the importance of continued public health efforts to expand awareness and education about rabies prevention and control, responsible animal ownership, routine rabies vaccination, appropriate personal protective equipment for barrier protection when performing laboratory procedures with potentially infected animals, and consistent interdisciplinary communication.

**Summary**

**What is already known about this topic?**

Captive mammals in zoological parks that are not completely excluded from rabies host species can become infected. Translocation of captive animals infected with rabies is responsible for spread of rabies in the United States.

**What is added by this report?**

Rabies virus has not previously been reported in tamanduas (anteaters). A rabies-infected tamandua was translocated from Virginia to Tennessee, exposing multiple persons. Postexposure vaccination was received by 13 persons. No human cases occurred.

**What are the implications for public health practice?**

Persons who routinely work with animals in areas where rabies is endemic should consider rabies preexposure vaccination. Efforts to expand rabies prevention and control awareness, responsible animal ownership, routine rabies vaccination, and interdisciplinary communication are important.

## Footnotes

* 45 C.F.R. part 46.102(l)(2), 21 C.F.R. part 56; 42 U.S.C. Sect. 241(d); 5 U.S.C. Sect. 552a; 44 U.S.C. Sect. 3501 et seq.
